# Advancing the “sexual” in sexual and reproductive health and rights: a global health, gender equality and human rights imperative

**DOI:** 10.2471/BLT.23.291227

**Published:** 2023-12-14

**Authors:** Tedros Adhanom Ghebreyesus, Pascale Allotey, Manjulaa Narasimhan

**Affiliations:** a World Health Organization, Geneva, Switzerland.; b Department of Sexual and Reproductive Health and Research, World Health Organization, Avenue Appia 20, 1211 Geneva 27, Switzerland.

As one aspect of population health, the global interest in women’s health has focused largely on maternal health and has implicitly ignored other aspects of women’s sexual and reproductive health and well-being. Despite this focus, maternal mortality remains unacceptably high,^1^ reflecting an indictment of our health systems, with insufficient access to services, uneven quality of care and an over-stretched health workforce. However, women’s health extends beyond motherhood, and across their lives the issues stemming from physiological, psychological, or societal factors significantly affect their well-being and quality of life. Often overlooked are sexual health needs of women including menstruation, endometriosis, vulvodynia, dyspareunia and peri- to post-menopause, as conditions that are often normalized to womanhood. Sexual health needs of men are also often overlooked, including infertility, premature ejaculation, impotence, and psychosexual concerns. Furthermore, millions are affected by sexually transmitted infections, including human immunodeficiency virus (HIV), reproductive tract infections and cancers of the reproductive system of women (cervical, breast, uterine and ovarian cancers) and men (prostate and testicular cancers). Defining the potential challenges and health risks at every life stage allows for proactive health-care interventions, education and support systems that promote positive sexual health outcomes throughout a person’s life.

Sexual health is fundamental to the overall health and well-being of all people, to the dignity of the individual and to the social and economic development of communities and countries.^2^ Sexual health of women and girls, and gender-diverse individuals is politicized. Violations of human rights in the context of sexual health are embedded in hierarchical structures of gender, generation, lineage, race, class and caste, in which more powerful or privileged people control the bodies and emotions of the less powerful. People with diverse sexual orientations and gender identities often face stigma and discrimination, and, in some countries, are subjected to extreme violence and are criminalized. Social exclusion of gender-diverse populations reduces their access to sexual and reproductive health care. There is a lack of evidence and capacity to address specific sexual health needs, and fear of discriminatory treatment by health and care workers confound this problem of access. There is a general paucity of research on the sexual health of transgender individuals, especially concerning sexual coercion, sexual and intimate partner violence, relationship quality, sexual risk behaviours and structural barriers to sexual health care access. Further research is also required to address important aspects of gender-affirming care. The focus on predominantly cisgender and heterosexual populations also means that evidence and guidance around key biomedical and health services, as well as health outcomes, are lacking.

The World Health Organization defines sexual health as a state of physical, emotional, mental and social well-being related to sexuality; it is not merely the absence of disease, dysfunction or infirmity.^3^ Sexual health requires a positive and respectful approach to sexuality and sexual relationships, as well as the possibility of having pleasurable and safe sexual experiences, free of coercion, discrimination and violence.^4^ For sexual health to be attained and maintained, the sexual rights of all individuals must be respected, protected and fulfilled.^5^

Sexual health is intrinsic to overall health and essential for thriving individuals, families and societies.^6^ Upholding sexual health is a moral obligation. Immense suffering is caused when people lack bodily autonomy; control over their fertility; and the freedom to experience safe, consensual and pleasurable sexual relationships. Sexual health is also inextricably linked with numerous health issues prioritized in global and national policy agendas, including HIV; maternal and newborn health; and the human rights agenda on sexual orientation, gender identity and expression, and sex characteristics. Investments in sexual and reproductive health can have a ripple effect, saving people’s lives while also improving health and well-being.^7^

Furthermore, sexual health is critical for gender equality and people’s empowerment, as recognized in the sustainable development goals.^8^^,^^9^ When people lack control over sex and reproduction, they cannot fully participate in social, economic and political spheres. Sexual health even impacts environmental sustainability. Slowing unsustainable population growth by investing in family planning and education reduces pressures on natural resources and helps break cycles of poverty.

The obstacles to advancing sexual health as part of a comprehensive approach to sexual and reproductive health and rights are deeply rooted in political dynamics, social values and gender inequalities. Steps to make universal sexual and reproductive health and rights a reality (Box 1) can make a difference if applied ambitiously and sustainably. With persistent effort on all fronts, we can foster societies where all people can experience their sexuality safely, positively and with dignity. The health and human rights imperatives are clear – it is time for the global health community to unite around a bold agenda to affirm and secure sexual health as part of sexual and reproductive health and rights for all.

Box 1 **Steps to make universal sexual and reproductive health and rights a reality**

1. Political leaders at all levels must champion sexual health as part of sexual and reproductive health to counteract conservative opposition. Sexual and reproductive health and rights affirm universal values that cut across religious, partisan and cultural divides. Leaders should frame them persuasively as mainstream health, development and human rights concerns, not fringe social issues.
2. Policy-makers must enact progressive laws and policies to expand access to comprehensive sexual and reproductive health services. Governments should fund public health programmes that integrate sexual and reproductive health within primary health care. Countries must repeal laws that criminalize homosexuality, sex work and HIV transmission, which drive at-risk, underserved individuals and communities away from health services.
3. Public health professionals need evidence-based strategies to reach the most excluded people and communities with quality care and information. Creative, equitable solutions, including through self-care and digital interventions, are required to serve remote areas, marginalized communities, unmarried youth and other underserved populations. Successful models from programmes showing health and rights gains through integrated services should be applied more widely.
4. Civil society and affected communities must mobilize to demand services, promote rights and reduce stigma. Youth activists, feminist advocates, HIV groups and others have catalysed progress through transnational campaigns and participatory health initiatives tailored to local contexts. However, broader citizen engagement and public pressure is still needed to influence political action.
5. Global leadership and funding are essential. International institutions should ensure sexual health is integrated within health, development and human rights frameworks. Foreign aid donors must dedicate resources to match the scale of needs in poor nations. Philanthropies also have potential to fund innovative solutions.

HIV: human immunodeficiency virus.

The *Bulletin of the World Health Organization* has issued a call for papers for a 2024 theme issue on sexual health and well-being,^10^ providing an opportunity to improve the dialogue around a crucial aspect of all of our lives.
